# Vaginal Probiotic *Lactobacillus crispatus* Seems to Inhibit Sperm Activity and Subsequently Reduces Pregnancies in Rat

**DOI:** 10.3389/fcell.2021.705690

**Published:** 2021-08-13

**Authors:** Ping Li, Kehong Wei, Xia He, Lu Zhang, Zhaoxia Liu, Jing Wei, Xiaomei Chen, Hong Wei, Tingtao Chen

**Affiliations:** ^1^School of Life Sciences, Institute of Translational Medicine, Nanchang University, Nanchang, China; ^2^Department of Obstetrics and Gynecology, The Ninth Hospital of Nanchang, Nanchang, China; ^3^Department of Obstetrics and Gynecology, The Second Affiliated Hospital of Nanchang University, Nanchang, China; ^4^Institute of Precision Medicine, The First Affiliated Hospital, Sun Yat-sen University, Guangzhou, China

**Keywords:** *Lactobacillus crispatus*, vaginal microbiome, sperm, animal model, reproductive health, offspring

## Abstract

**Background:**

The vaginal microbiota is associated with the health of the female reproductive system and the offspring. *Lactobacillus crispatus* belongs to one of the most important vaginal probiotics, while its role in the agglutination and immobilization of human sperm, fertility, and offspring health is unclear.

**Methods:**

Adherence assays, sperm motility assays, and Ca^2+^-detecting assays were used to analyze the adherence properties and sperm motility of *L. crispatus* Lcr-MH175, attenuated *Salmonella typhimurium* VNP20009, engineered *S. typhimurium* VNP20009 DNase I, and *Escherichia coli* O157:H7 *in vitro.* The rat reproductive model was further developed to study the role of *L. crispatus* on reproduction and offspring health, using high-throughput sequencing, real-time PCR, and molecular biology techniques.

**Results:**

Our results indicated that *L. crispatus*, VNP20009, VNP20009 DNase I, and *E. coli* O157:H7 significantly inhibited the sperm motility *in vitro via* adversely affecting the sperm intracellular Ca^2+^ concentration and showed a high adhesion to sperms. The *in vivo* results indicated that *L. crispatus* and other tested bacteria greatly reduced the pregnancy rates, but *L. crispatus* had a positive effect on maternal health and offspring development. Moreover, the transplantation of *L. crispatus* could sustain a normal bacterial composition of the vaginal microbiota in healthy rats and markedly reduced the expression of uterine inflammatory factors (toll-like receptor-4/nuclear factor kappa-B, tumor necrosis factor-α, production of interleukin-1β, etc.) and apoptosis factors (Fas Ligand, Bcl-2-associated X protein/B cell lymphoma-2, etc.) compared with the other tested strains.

**Conclusion:**

Our study demonstrated that the vaginal probiotic *L. crispatus* greatly affected the sperm activity and could also reduce pregnancies through its adhesion property, which might account for some unexplained infertility. Therefore, more caution should be paid when using *L. crispatus* as a vaginal viable preparation in women of child-bearing age, especially for women whose partners have abnormal sperms.

## Introduction

Multicellular organisms co-evolved with a complex coalition of acellular viruses, bacteria, fungi, and protist, collectively known as the microbiota (Belkaid and Hand, 2014). In mammals, the microbiota that reside in the vagina may interact with the host in a complex series of physiologic processes (Chen et al., 2017), including development, metabolism, and immune function (Cohen, 2016; Halmos and Suba, 2016). In recent years, there is an increasing attention on the vaginal microbiota specific to female reproductive and offspring health (Chen et al., 2017), and published studies have indicated that the vaginal microbiota plays a crucial role not only in health and disease but also in fertilization and pregnancy (Younes et al., 2018).

The human vagina is an important channel for women to connect the internal and the external genitalia, for sexual intercourse, for menstrual blood to flow out, and for fetal delivery (Pellati et al., 2008). The vaginal discharge provides nutrients for the microbiota, among which the healthy vaginal microbiota is mainly dominated by *Lactobacillus* spp., such as *Lactobacillus iners*, *Lactobacillus crispatus*, *Lactobacillus jensenii*, and *Lactobacillus gasseri* (Ling et al., 2010). They exert important promoting effects in maintaining the health condition of the female genital system (Petrova et al., 2015). *L. crispatus* appears to be substantially superior to the other *Lactobacillus* species (Shipitsyna et al., 2013) for its benefits of promoting the stability of normal vaginal microbiota (Verstraelen et al., 2009). Apart from that, it also has some other probiotic properties, including inhibiting various genital tract pathogenic bacteria (e.g., *Gardnerella vaginalis*) and fungi (e.g., *Candida albicans*) (Reid, 2017), increasing the antibiotic sensitivity, and reducing the risk of sexually transmitted diseases such as HIV (Tuddenham and Ghanem, 2017). These properties of *L. crispatus* can be attributed to the production of bactericidal substances (e.g., lactic acid and hydrogen peroxide) (Gong et al., 2014), ecological niche occupation (Srinivasan et al., 2009; Sgibnev and Kremleva, 2017), and immunomodulatory effects (Reid, 2017). Additionally, *L. crispatus* can prevent the colonization of pathogens *via* adhering to host cells (Younes et al., 2018), and the strong adherent effect of *L. crispatus* is considered to be more beneficial to the human body (Rizzo et al., 2015), but few studies focus on the possible negative effects caused by this characteristic.

As we have known, infertility is the condition of being unable to produce offspring and is defined by the World Health Organization as “a disease of the reproductive system defined by the failure to achieve a clinical pregnancy after 12 months or more of regular unprotected sexual intercourse” (Zegers-Hochschild et al., 2017). The common causes of infertility include genetic, immunological, and anatomical reasons (Mandar et al., 2017). Notably, the microbiological factors account for about 15% of overall couple infertility, and *Escherichia coli* has been implicated as one of the most common ones (Monteiro et al., 2018; Dutta et al., 2020). As early as in 1931, Rosenthal L demonstrated the agglutination effect of *E. coli* on sperm; subsequent studies also proved its immobilization effect. Both of the effects can remarkably reduce the vitality of sperm and cause infertility (Answal and Prabha, 2018). As one of the dominant species of the vaginal microbiota, the influence and the mechanism of *L. crispatus* on fertility are still unclear.

Our previous work indicated that some of the *Lactobacillus* spp. studied (*L. crispatus*, *Lactobacillus acidophilus*, *Lactobacillus salivarius*, *Lactobacillus helveticus*, and *L. gasseri*) could effectively adhere to sperm and greatly reduce the sperm motility. Of these, *L. crispatus* could greatly reduce the ability of sperm to penetrate the viscous medium *in vitro*, which suggest that this resident may influence the reproductive outcomes *via* some ways (Wang et al., 2020). Hence, the effect of *L. crispatus* as the vaginal probiotic product should be reconsidered, especially for some couples whose men are in a sub-fertile state.

In the present study, we verified whether *L. crispatus* had the potential to inhibit sperm motility *in vitro*, and we also evaluated the ability of *L. crispatus* in regulating the vaginal microbiota and affecting the reproductive results as a new vaginal agent both in maternal and offspring rat compared with other pathogenic bacteria. This study serves as a helpful warning and a theoretical basis for women preparing for a pregnancy which requires *L. crispatus* as a vaginal probiotic agent. Simultaneously, it is significant to further study and evaluate the probiotic activity of *L. crispatus* while avoiding the reduction of sperm motility.

## Materials and Methods

### Semen Sample Collection and Treatment

Semen samples of male subjects were collected from March 2019 to June 2019 after signing the informed consent (age = 22–38 years, without leukocytospermia), which was approved by the Institutional Ethics Committee on human subjects of The Second Affiliated Hospital of Nanchang University (ChiCTR2000036420). Donors who had been proven to be fertile and had normal sperm parameters according to the WHO criteria (Cao et al., 2011) were recruited. The subjects with abnormal karyotype and genital injuries were excluded. The subjects masturbated and ejaculated the semen into a 15-ml sterile collection tube. The semen samples were examined under a microscope and incubated in 37°C water bath for 25–45 min until they were liquefied, and then the purified supernatant sperms were harvested by direct swim-up in human tubal fluid (HTF) medium (Millipore) for subsequent *in vitro* experiments (Alasmari et al., 2013). The semen samples obtained on the same day were used immediately, and the sampling process continued until the end of the *in vitro* experiment.

### Bacterial Strain Treatment

VNP20009 (ATCC 202165, an attenuated *Salmonella typhimurium* VNP20009), VNP20009 DNase I (an engineering bacteria, which was constructed by electrically transforming plasmid pLIVE-DNase I into attenuated *S. typhimurium* VNP20009, DNase I sequence number NM_005223.3, which was generated at Kingsy Biotechnology Co., Chen et al., 2019), *E. coli* O157:H7 (ATCC 12806), and *L. crispatus* (*L. crispatus* Lcr-MH175, number CGMCC 15938, identified by Harbin Meihua Biotechnology Co., Ltd., Harbin, Heilongjiang, China) were stored at −80°C. These strains were cultivated in a proper medium [*L. crispatus* was cultivated in De Man, Rogosa, Sharpe Broth (MRS, Hopebio, HB0384-1, Qingdao); VNP20009 and *E. coli* O157:H7 were cultivated in Lysogeny Broth (LB, Hopebio, HB0384-1, Qingdao), and VNP20009 DNase I was cultivated in LB medium containing 50 μg/ml ampicillin] for 24 h in an incubator at 37°C and activated twice; the bacterial growth density of each strain was approximately 10^9^ CFU/ml. Taking 50 μl, 10^8^ sperms/ml purified sperm samples was mixed with HTF medium containing different bacterial strains in equal volumes, and the number ratio of sperm to bacteria was 1:10. Subsequently, the mixed samples were incubated in an incubator at 37°C and 5% CO_2_ for different times. The experiment was carried out according to the following procedures.

### Adhesion Assays

After incubation for 2, 4, and 6 h, the mixture of the sperm samples and bacterial strains was washed with sterile HTF solution four times. Briefly, the mixed samples were centrifuged at 400 × *g* for 5 min; the supernatant was removed and resuspended with 100 μl sterile HTF solution. A mixture of 10 μl was placed on a slide to dry, then fixed with methanol for 30 min, stained using the Gram staining procedure, and examined under a ×40 objective on an optical microscope (Olympus BX63). The number of adhesion bacteria in 100 sperms was measured from 25 microscopic fields which were randomly selected. Each adhesion test was repeated three times.

### Assessment of Sperm Motility

A computer-assisted sperm analysis system (WLJY-9000, WeiLi Co., Ltd., China) was used to detect and analyze sperm motility after incubation with the tested bacteria for 2, 4, and 6 h. The sperm parameters related to total motility, progressive motility, linear velocity, and immotile sperm were measured. At least 200 sperms were examined in each trial. Each sperm motility assay was performed three times.

### Sperm Penetration Assay

According to the published experimental method of Ivic et al. (2002), a 1% (w/v) methylcellulose solution was used to simulate the viscous environment of the female reproductive tract. Methylcellulose was prepared using HTF medium and inhaled into a flat capillary tube with a length of 7.5 cm and an inner diameter of 1.0 mm, one end of which was sealed with plasticine. Human sperms and tested bacteria were incubated in an incubator at 37°C, 5% CO_2_, for 2 h, and the open end of the capillary tube was inserted into the incubation mixture. After 1 h, the tubes were removed, and the penetration of methylcellulose was recorded using a Leica DM2500 upright microscope. The average number of sperms in the three sperm fields (10×) was counted at 1 and 2 cm from the base of the tube, respectively.

### Measurement of Intracellular Ca^2+^ Concentrations in Sperm Samples

The Ca^2+^ signaling in human sperm was examined by sperm [Ca^2+^]_*i*_ imaging (Nash et al., 2010). After the human sperms were loaded with 5 μM Fluo-4 AM (Molecular Probes, United States) and 0.05% pluronic F-127 (Molecular Probes, United States) at room temperature in the dark for 30 min, these were then washed in HTF medium. The washed sperms were loaded on a 96-well cell culture dish (Nest Biotechnology Co., Ltd.) and quickly added with the tested bacteria, respectively. A Polychrome V chromator (TILL Photonics GmbH, Germany) was used to image the sperm [Ca^2+^]_*i*_ signaling. The generated 488-nm excitation light and the emissions (515–565 nm) were bandpass-filtered (HQ540/50, Chroma), which were collected with a cooled CCD camera (CoolSNAP HQ, Roper Scientific). The mixture was recorded for 200 s (50 ms of exposure time and 2 s of time interval).

### Animal Set

Sexually mature and healthy 10- to 11-week-old male (*n* = 18, 313.3 ± 21.3 g) and 9- to 10-week-old female Sprague–Dawley (SD) rats (*n* = 72, 213.3 ± 21.3 g) were purchased from Nanchang Royo Biotech Co., Ltd. The animals were habituated to the animal facility for 2 weeks before the experiments under a 12-h light/dark cycle, with temperature of 21 ± 1°C and humidity of 55 ± 10%. Food and water were given *ad libitum*.

### Reproductive Studies

In the first experimental set, the animals were randomly divided into five groups, i.e., control group (C group, *n* = 8), VNP20009 group (V group, *n* = 8), VNP20009 DNase I group (VD group, *n* = 8), *E. coli* O157:H7 group (E group, *n* = 8), and *L. crispatus* group (L group, *n* = 8). For the C group, the rats were held in a supine position during the application, and then the medical absorbable gelatine sponges (0.5 cm × 0.5 cm) which were soaked in 50 μl of phosphate-buffered saline (PBS) were delivered into the vaginal cavities of the rats once a day; the entire administration lasted 25 days. Similar to the C group, the V group, VD group, E group, and L group were treated with the bacterial strains of VNP20009, VNP20009 DNase I, *E. coli* O157:H7, and *L. crispatus*, respectively (the concentration of each bacterial strain was about 5 × 10^4^ CFU/ml). All females were immediately cohabited with males in a ratio of 4:1 after the sixth administration. When mating was confirmed by the presence of a vaginal plug or a pregnancy outcome, the females were separated from the males. On the 1st day, the administration of bacteria was stopped for 1 day, and the vaginal secretions in all groups were collected from donor dams with 200 μl sterile saline, and the entire contents of the pipette were expelled into the tube, which was conserved at −80°C for further use. On the 21st day, all the females were separated from the males and killed; their vaginas were collected for subsequent experiments.

### Offspring Health Studies

In this experiment, the animals were randomly divided into three groups, i.e., control group (C group, *n* = 8), wide-spectrum antibiotic group (ABX group, *n* = 12), and *L. crispatus* group (L group, *n* = 12). For the C group, medical absorbable gelatine sponges (0.5 cm × 0.5 cm) were soaked in 50 μl PBS, and then delivered into the vaginal cavities each day as previously described. Similar to the C group, the L group was treated with the bacterial strain of *L. crispatus* whose concentration was about 5 × 10^4^ CFU/ml, and the ABX group was treated with a wide-spectrum antibiotic [50-μl mixture of ampicillin (100 mg/ml), metronidazole (100 mg/ml), neomycin (100 mg/ml), and vancomycin (50 mg/ml)]. All females were immediately cohabited with males in the ratio of 4:1 after the sixth administration. On the 21st day, all the females were separated from the males, and they waited for parturition. When the pups were all delivered, six pups at postnatal day (P) 7 were randomly selected from each group for their colon and vaginal samples to be collected. The body weight of the remaining pups was measured from P 7 to P 42. After 2 weeks, the pups underwent the open-field test and the pole test. Finally, all the pups were sacrificed after 6 weeks; their tissues were collected and stored at −80°C for further use.

### Fetal Health Assessment

The determination of live, dead, absorbed, and malformed fetus observed the following criteria: a live fetus showed a fleshy red color, fully formed, with natural movement, with motor response to mechanical stimulation, with a red placenta, large, and with its heart beating. A dead fetus demonstrated a grayish-red or dark purple color, fully formed, with recognizable toes of the limbs, with a body weight of ≤0.8 g, with no natural movement or response to mechanical stimulation, with the placenta grayish-red in color, and slightly smaller. An absorbed fetus revealed a dark purple or pale white color, not fully formed, with visible limb buds but no toes, with autolysis or softening, with no natural movement, with the placenta dark purplish red in color, and small. A malformed fetus evidenced the toes of the limbs being well formed, but the head, limbs, or tail was missing.

### Behavioral Assessment

The pole test and the open-field test were performed to evaluate the development of the offspring. The pole test was used to determine the ability of movement balance and body coordination (Meligy et al., 2019), and the open-field test was used to evaluate the capacity for exploratory activities (Vakhnin and Briukhin, 2014). In the pole test, the rats were placed facing upward at the top of a rough wooden pole. Each rat got training (3–5 min/day) in the pole-descending behavior for 3 days, and only those rats revealing a time <18 s in the pre-test trial were used to participate in the formal trials. In the open-field test, each rat was individually placed in a chamber of size 100 cm × 100 cm (16 peripheral and nine internal). Each rat was placed in the center of the area to acclimate for 10 min, and then its behavioral parameters were monitored for 10 min with the ethovision video tracking software (Noldus, Wageningen, Netherlands). At the end of each trial, the equipment was cleaned with 75% ethanol to minimize odor interference to the next rat.

### High-Throughput Sequencing Analysis

The genomic DNA of vaginal secretions was extracted using a DNA extraction kit (Tiangen Biotech Co., Ltd., Beijing, Cat#DP302-02), and the V4 region of the 16S rRNA genes was amplified using the primer 515F/806R (Walters et al., 2016). An Illumina HiSeq 2000 platform was applied to sequence these PCR products (GenBank accession number: PRJNA637679) (Edgar, 2013). Sequence analysis was subsequently processed using the UPARSE software package (version 7.0.100). Then, the QIIME software (version 1.9.1) was used to analyze the α diversity [indexes of observed operational taxonomic units (OTUs), Chao1, Shannon, Simpson, ACE, and goods coverage], β diversity [principal component analysis, principal coordinate analysis, and non-metric multidimensional scaling (NMDS)], differently abundant taxa identifications (Metastats and LEfSe analysis), and cluster analysis, and partial least squares discriminant analysis was performed using SIMCA-P software (version 11.5).

### Histopathological Analysis

After dissecting the maternal rats, the fresh uterine tissue was put into the prepared 4% paraformaldehyde for fixation. Dehydration and transparent treatment were carried out, paraffin embedding was carried out, and the tissue was sliced into 5-μm segments. Before staining, these were rehydrated by xylene and ethanol for 5–6 min and washed three times for final hematoxylin and eosin (H&E) staining. Rabbit anti-Fas Ligand (FasL) antibody [1:1,000, Cell Signaling Technology (CST), Cat#ab15285] was used for immunohistochemical staining. Then, an antigen repair was performed, and the subsequent antibody binding and color rendering were completed according to the antibody instructions.

### QRT-PCR and ELISA

In order to evaluate the mRNA expression level of cytokines associated with inflammation in female rat uterine tissues, the total RNA extracted from uterine tissues was reverse-transcribed into cDNA using a commercial reverse transcription kit (Takara, RR047A) (Sawant et al., 2021). Then, qRT-PCR was performed using Takara TB Green^®^ Premix Ex Taq^TM^ (Tli RNaseH Plus) Kit (Takara, RR420A) to detect the expressions of tumor necrosis factor α (TNF-α), interleukin 6 (IL-6), interleukin 1β (IL-1β), and the housekeeping gene glyceraldehyde 3 phosphate dehydrogenase (GAPDH). After normalization against GAPDH, the relative amount of target gene transcripts in each cDNA sample was measured (Wang and Liu, 2020). The relative expression levels of these cytokines were analyzed by the 2^−ΔΔ*Ct*^ method. The primer sequences were as follows:

TNF-α: forward primer – 5′-GTGGAACTGGCAGAAGA GGCA-3′, reverse primer – 5′-AGAGGGAGGCCATTTGG GAAC-3′;
IL-6: forward primer – 5′-GAAATCGTGGAAATGAGA- 3′, reverse primer – 5′-GCTTAGGCATAACGCACT-3′;
IL-1β: forward primer – 5′-GTGTCTTTCCCGTGGAC CTTC-3′, reverse primer – 5′-TCATCTCGGAGCCTGTA GTGC-3′;
GAPDH: forward primer – 5′-CTCGTGGAGTCTACTGG TGT-3′, reverse primer 5′-GTCATCATACTTGGCAG GTT-3′.

The collection of serum samples of female rats was *via* retro-orbital hemorrhage. For the determination of inflammatory cytokine levels in serum samples, ELISA kits for TNF-α (Cloud Clone Crop, United States, Cat #SEA133Mu), IL-6 (Cloud Clone Crop, United States, Cat #SEA079Mu), and IL-1β (Cloud Clone Crop, United States, Cat #SEA563Mu) were used. The inflammatory factors in serum bound to the biotinylated anti-rat monoclonal antibody and the immune complex could be formed after adding the secondary antibody, which combined to streptavidin labeled with horseradish peroxides. The addition of tetramethylbenzidine can make it colored, and the expression of the concentration of inflammatory factors in serum could be calculated according to its OD value.

### Western Blotting Analysis

Total proteins were extracted from lysed tissues using a cell lysis buffer (Solarbio, R0010) containing protease inhibitors (MedChemExpress, HY-K0010). After the protein samples were denatured in boiling water, sodium dodecyl-sulfate polyacrylamide gel electrophoresis was completed using the electrophoresis apparatus. At this time, the total proteins in the samples were separated and arranged in the gel, and then the separated proteins were transferred to the polyvinylidene fluoride membrane by wet transfer. Subsequently, the membranes were sealed in 5% milk to block the non-specific site, and this was incubated overnight with the corresponding primary antibody at 4°C and then incubated with the secondary antibody conjugated with horseradish peroxidase (Chen et al., 2019). Finally, the membranes were observed and photographed using a chemiluminescence detection system (Tanon-5200, 20T12NPFLI6-10111). The primary antibodies including anti-β-actin (1:1,000, rabbit, CST, Cat#4970S), anti-transforming growth factor β1 (TGF-β1) (1:1,000, rabbit, CST, Cat#3711), anti-Bcl-2-associated X protein (Bax) (1:1,000, rabbit, CST, Cat#5023), anti-B cell lymphoma-2 (Bcl-2; 1:1,000, rabbit, CST, Cat#3498), anti-toll-like receptor 4 (TLR-4; 1:1,000, mouse, Santa Cruz, Cat#sc-293072), anti-phosphorylated nuclear factor kappa-B p65 (p-p65) (1:1,000, rabbit, Abcam, Cat#ab86299), anti-p65 (1:1,000, rabbit, CST, Cat#8242S), anti-zonula occludens-1 (ZO-1; 1:1,000, rabbit, CST, Cat#5406S), and anti-Occludin (1:1,000, rabbit, CST, Cat#91131).

### Statistical Analysis

All values were expressed as mean ± standard deviation (SD). The differences of the experimental data between the control and samples were assessed with one- or two-way analysis of variance (ANOVA). Data handling, analyses, and graphical representations were performed using the GraphPad Prism software, version 7.0 (San Diego, CA, United States; RRID: SCR_004812). A *P*-value < 0.05 was regarded as statistically significant.

## Results

### Effect of *L. crispatus* on Human Sperm Motility Compared With Other Strains

To evaluate whether the probiotic *L. crispatus* had a better health effect compared to potential pathogens, we used *E. coli* O157:H7, a pathogenic bacterium capable of impairing sperm viability, as a positive control. In addition, we engineered the *S. typhimurium* VNP20009 DNase I strain, which has plasmid-containing DNase I to express DNase I toxin to harm the sperm activity, and *S. typhimurium* VNP20009 was used to serve as a control for *S. typhimurium* VNP20009 DNase I strain. Therefore, the three types of pathogenic bacteria, VNP20009, VNP20009 DNase I, and *E. coli* O157:H7, were compared with the probiotic *L. crispatus* to investigate their effects on sperm activity.

**FIGURE 1:**
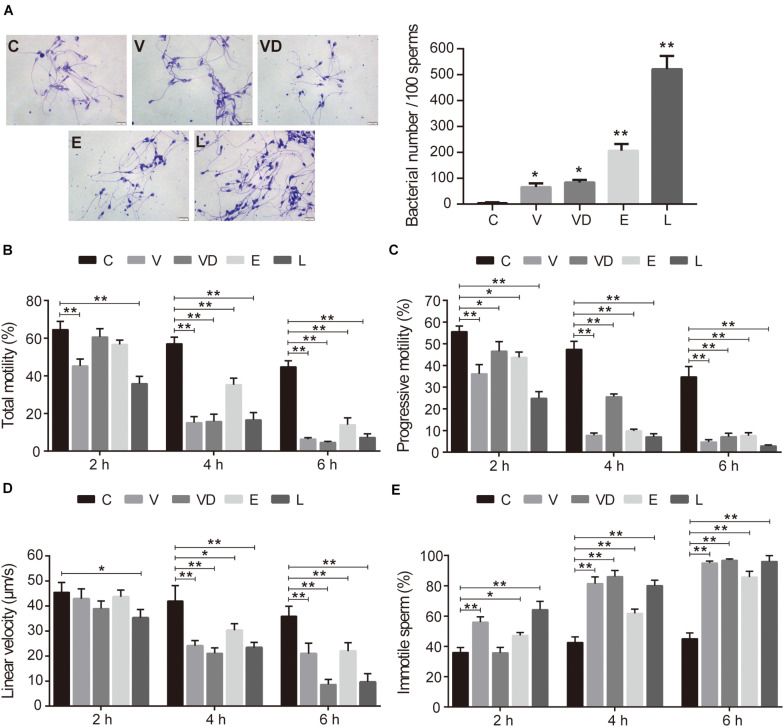
The effect of tested bacteria on sperm motility and the adhesion to sperm *in vitro*. **(A)** The gram stain results (magnification, ×100) and adhesion numbers of PBS, VNP20009, VNP20009 DNase I, *E. coli* O157:H7, and *L. crispatus*. The initial sperm/bacteria ratio was 1:10, and the bacteria were available in-house. Human ejaculated sperm were incubated with a single species of bacteria in HTF medium in an incubator at 37°C and 5% CO_2_ for 2, 4, and 6 h, respectively. The motion parameters including **(B)** total motility, **(C)** progressive motility, **(D)** linear velocity, and **(E)** immotile sperm were analyzed by computer-assisted sperm analysis. For exactly assessing the sperm viability, a minimum of 200 sperms were counted for each assay. The data represent means ± SD of three independent experiments. *P < 0.05, **P < 0.01. SD, standard deviation; C, control; V, VNP20009; VD, VNP20009 DNase I; E, *E. coli* O157:H7; L, *L. crispatus*.

After conducting adherence assays, we found that all tested bacteria could adhere to sperm, with adhesive indices of approximately 65, 83, 206, and 521 per 100 sperms for VNP20009, VNP20009 DNase I, *E. coli* O157:H7, and *L. crispatus*, respectively (Figure 1A). When analyzing the sperm motility, our results indicated that the adhered bacteria significantly decreased all parameters, reducing the total sperm motility (<40%), progressive motility (<30%), and linear velocity (<40 μm/s). In fact, an increase in the immotile sperm was also observed (>60%) when compared with the control at 4 and 6 h (Figures 1B–E). It is important to mention that *L. crispatus* could efficiently adhere to sperm and reduce the sperm motility *in vitro*.

### Reduction of Intracellular Ca^2+^ Concentration *in vitro*

**FIGURE 2:**
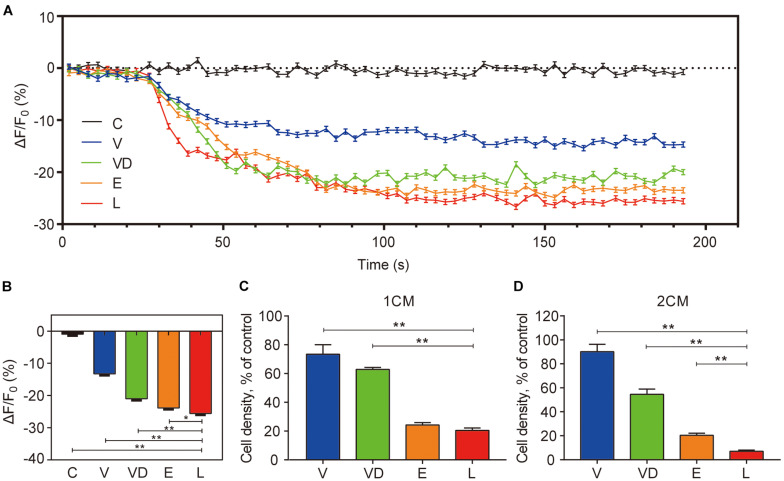
The effect of tested bacteria on the sperm of the intracellular Ca^2+^ concentration ([Ca^2+^]_*i*_) and the ability of penetration into viscous medium. **(A)** Sperm [Ca^2+^]_*i*_ was monitored after loading sperms with 5 μM Fluo-4 AM, and the fluorescence intensity of the sperm head was visualized and detected before and after adding the different species of bacteria. **(B)** The statistical analysis of the effects of different species of bacteria on sperm [Ca^2+^]_*i*_ at the time frame of 120 s as indicated in **(A)**. Human sperms were treated with phosphate-buffered saline as control and were exposed to different species of bacteria to assess the penetrating ability of sperms into a viscous medium. Cell density (percentage of control) of **(C)** 1 cm and **(D)** 2 cm into the methylcellulose are shown. The initial sperm/bacteria ratio was 1:10. The data represent means ± SD of three independent experiments. *P < 0.05, **P < 0.01. SD, standard deviation; C, control; V, VNP20009; VD, VNP20009 DNase I; E, *E. coli* O157:H7; L, *L. crispatus*.

As previously reported, [Ca^2+^]_*i*_ can regulate the chemotactic behavior of sperm and is pivotal to its function (Publicover et al., 2008). Thus, we further studied the effect of adhered bacteria on [Ca^2+^]_*i*_. Our results indicated that the adhered bacteria caused a reduction of human sperm [Ca^2+^]_*i*_ within 1 min, and L group had the lowest sperm [Ca^2+^]_*i*_ compared with the other groups at 2 min (Figures 2A,B). As shown in Figures 2C,D, the mean sperm numbers of penetrating 1% methylcellulose medium at 1 and 2 cm away from base of the tube for the controls were 489 and 166, respectively. VNP20009 DNase I and *E. coli* O157:H7 reduced 50 and 80% of the sperm numbers compared with the control group at 2 cm, while *L. crispatus* reduced the sperm numbers by approximately 90%.

### Effect of *L. crispatus* on Reproduction by Inhibiting Sperm Motility

**FIGURE 3:**
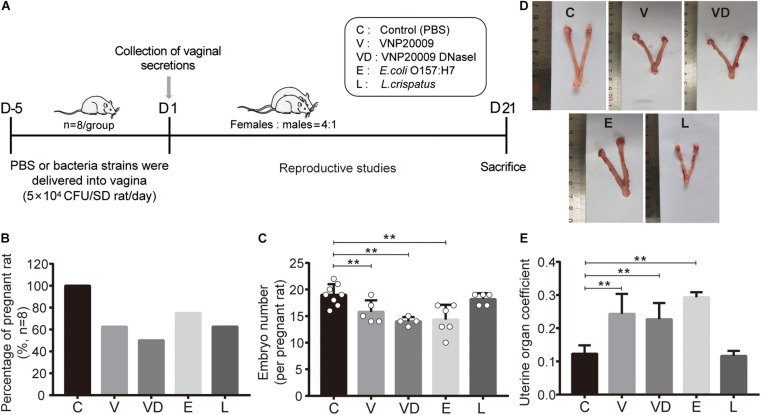
The inhibitory effect of *L. crispatus* on sperm reduced the pregnancy rates and had no reproductive impairment. **(A)** Scheme of experimental timeline and procedures. **(B)** Fertility outcome about pregnancy rates and **(C)** embryo number about the female rats of the indicated treatment groups. **(D)** Representative gross images and **(E)** organ coefficients of the uterus are shown in different treatment groups. The data represent means ± SD of eight to 10 animals. *P < 0.05, **P < 0.01. SD, standard deviation; C, control; V, VNP20009; VD, VNP20009 DNase I; E, *E. coli* O157:H7; L, *L. crispatus*.

A rat reproductive model was developed to study the potential adverse effect of tested bacteria on the conception of females. PBS, VNP20009, VNP20009 DNase I, *E. coli* O157:H7, and *L. crispatus* were transplanted into rat vaginal tracts, respectively (Figure 3A). After 25 days of continuous administration, our results showed that all the adhered bacteria resulted in a decrease of conception, and the pregnancy rates of the V, VD, E, and L group were 62.5, 50, 75, and 62.5%, respectively (Figure 3B). Comparing with the pregnant rats of the C group, the number of embryos in the V, VD, and E groups had a significant reduction, and there were also those that caused dead, absorbed, and malformed fetus, but the L group had no effect on the embryo health (Figure 3C and Supplementary Figure 1). The uterine morphology of unpregnant rats shown in the V, VD, and E groups were markedly swollen with a serious hyperemia, a bulbous expansion at the end of the uterine cavity, a thinner uterine wall containing a clear liquid in the lumen, and an increased uterine organ coefficient, but the morphology and the weight of the L group uterine were normal (Figures 3D,E), showing that VNP20009, VNP20009 DNase I, *E. coli* O157:H7, and *L. crispatus* exerted the inhibition of conception, whereas *L. crispatus* had no toxicity to the maternal uterine and offspring.

### Effect of Tested Bacteria on Uterine Tissue Proliferation and Inflammation

**FIGURE 4:**
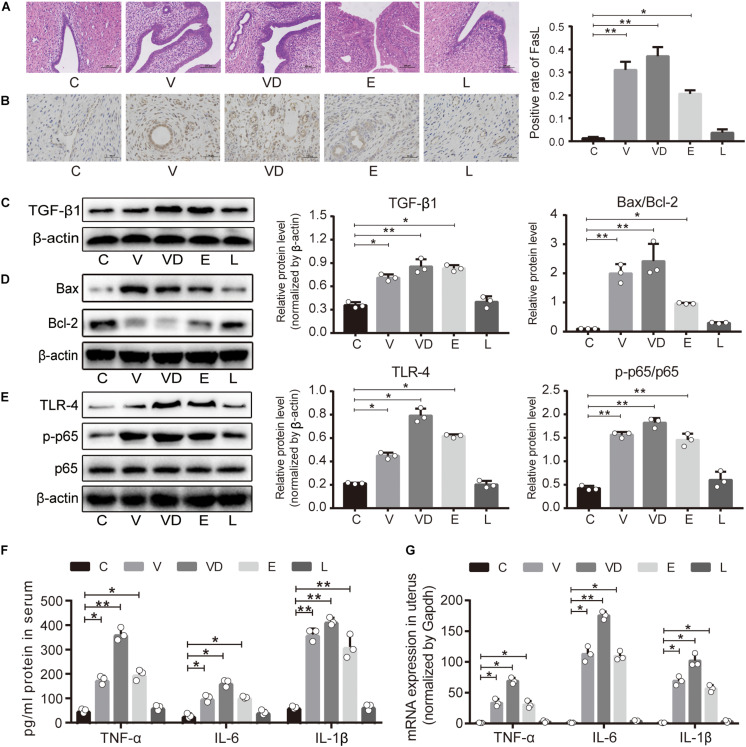
Treated vagina with *L. crispatus* had no influence on uterine impairment and vaginal inflammation. **(A)** Uterine histopathological examination was performed by H&E staining with different treatments; the bar represents 100 μm. **(B)** The immunohistochemistry images (left) of FasL expression were determined in the uterus from the indicated treatment groups; the graph on the right shows a positive rate counted in at least five different areas in the images; the bar represents 50 μm. The relative expression of **(C)** TGF-β1, **(D)** Bax/Bcl-2, and **(E)** TLR-4/NFκB was analyzed by western blotting in the vagina. **(F)** ELISA-based quantification of TNF-α, IL-6, and IL-1β levels in serum from the indicated treatment groups. **(G)** The mRNA expressions of TNF-α, IL-6, and IL-1β were determined by qPCR in the uterus. The data represent means ± SD of three independent experiments. *P < 0.05, **P < 0.01. SD, standard deviation; C, control; V, VNP20009; VD, VNP20009 DNase I; E, *E. coli* O157:H7; L, *L. crispatus*.

To further explore the mechanisms of adhered bacteria on conception, H&E staining was used, and it was observed that the uterine wall showed a clear demarcation of the different layers. Intact endometrial epithelial cells were observed in the C group and the L group without hyperemia and inflammatory cell infiltration, while a damaged uterine wall and inflammatory infiltration were observed in the V, VD, and E groups (Figure 4A). Furthermore, the immunohistochemistry results showed that the V, VD, and E groups clearly induced the production of Fas Ligand (FasL) (Figure 4B). Then, western blotting analysis was used to detect key proteins associated with uterine proliferation and inflammation, and significant increases of transforming growth factor β1 (TGF-β1), Bax/Bcl-2, toll-like receptor-4 (TLR-4), and p-p65/p65 ratio in the uterus were observed in the V, VD, and E groups (Figures 4C–E). Moreover, the mRNA expressions of tumor necrosis factor-α (TNF-α) and cytokines interleukin-6 (IL-6) and cytokines interleukin-1β (IL-1β) in the uterus were greatly downregulated compared with the C group, respectively (Figures 4F,G). For *L. crispatus*, it sustained the normal expressions of cytokines related to uterine tissue proliferation and inflammation compared with other pathogens.

### Effect of Tested Bacteria on the Composition of the Vaginal Microbiota

**FIGURE 5:**
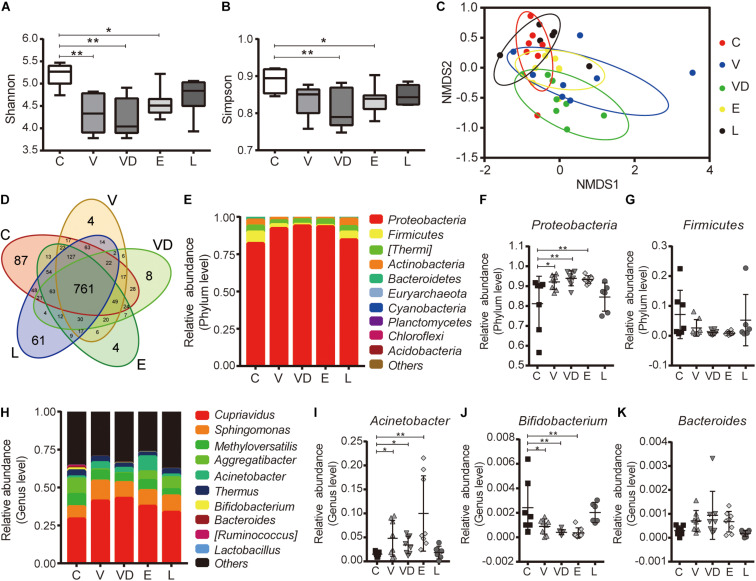
The effect of the tested bacteria on the vaginal microbiota diversity of rats. **(A)** Chao1 index and **(B)** Shannon index among the indicated treatment groups. **(C)** NMDS analysis of the indicated treatment groups. **(D)** Venn diagram of the vaginal microbiota among the indicated treatment groups. **(E)** Microbial composition at the phylum level. Relative abundance of **(F)** *Proteobacteria* and **(G)** *Firmicutes* of vaginal bacterial communities in phylum level among the indicated treatment groups. **(H)** Microbial composition at the genus level. Relative abundance of **(I)** *Acinetobacter*, **(J)** *Bifidobacterium*, and **(K)** *Bacteroides* of vaginal bacterial communities at the genus level among the indicated treatment groups. The data represent means ± SD of six to eight animals. *P < 0.05, **P < 0.01. SD, standard deviation; C, control; V, VNP20009; VD, VNP20009 DNase I; E, *E. coli* O157:H7; L, *L. crispatus*; NMDS, non-metric multidimensional scaling.

To determine the effect of bacterial transplantation on vaginal microbial composition, we performed 16S rRNA gene sequencing of DNA isolated from vaginal secretion samples from the C, V, VD, E, and L groups. As shown in Figures 5A,B, the alpha-diversity of microorganism (Shannon and Simpson) was higher in the C and L groups compared with the V, VD, and E groups. The NMDS analysis (beta diversity) indicated that the samples in the C and L groups were clustered together, while the samples in the V, VD, and E groups were diverged from the C group (Figure 5C). Besides this, the Venn diagram showed that 761 common OTUs were identified in all groups, among which 48 OTUs were identified in the C and L groups, 17 OTUs were identified in the C and V groups, 28 OTUs were identified in the C and VD groups, and 13 OTUs were identified in the C and E groups (Figure 5D). At different taxonomic levels, we found that the number of microbial groups contained in the V, VD, and E groups were reduced compared with the C and L groups (Supplementary Figure 2).

Furthermore, the microbial community structure was also changed at the phylum and genus levels (Figures 5E,H). At the phylum level, a higher abundance of *Proteobacteria* was observed in the V, VD, and E groups (C vs. V, *P* = 0.019; C vs. VD, *P* = 0.0055; C vs. E, *P* = 0.0081). The abundance of *Firmicutes* did not show significant differences (C vs. V, *P* = 0.3297; C vs. VD, *P* = 0.1024; C vs. E, *P* = 0.0867), but we could also find a decrease in the V, VD, and E groups (Figures 5E–G). We next examined microbiota at the genus level. The abundance of *Bifidobacterium* was reduced in the V, VD, and E groups (C vs. V, *P* = 0.0275; C vs. VD, *P* = 0.0029; C vs. E, *P* = 0.0024), corresponding to the change of *Firmicutes* at the phylum level, and a higher abundance of *Acinetobacter* (C vs. V, *P* = 0.0349; C vs. VD, *P* = 0.0278; C vs. E, *P* = 0.0018) and *Bacteroides* (C vs. V, *P* = 0.5224 C vs. VD, *P* = 0.1303; C vs. E, *P* = 0.6012) was observed in the V, VD, and E groups, also corresponding to the change of *Proteobacteria* at the phylum level, although without statistical significance in *Bacteroides* genus level (*P* > 0.05) (Figures 5I–K). In addition, the LEfSe and Metastats analysis indicated that some taxa abundances were also significantly changed in the V, VD, and E groups compared with the C and L groups, for example, the abundance of *Lactobacillaceae* was significantly increased in the L group (Supplementary Figures 3A,B).

### Effect of Vaginal Transplantation of *L. crispatus* on Offspring Health

To further assess whether *L. crispatus* mediated the spermicidal effect of bacteria on the offspring, we compared the offspring whose maternal vaginal tracts were transplanted with PBS, broad-spectrum antibiotics (to exclude the influence of vaginal microbiota on sperm), and *L. crispatus*. As presented in Supplementary Figure 4, ABX greatly reduced the overall microbial abundance including the observed species (C vs. ABX, *P* = 0.0010; ABX vs. L, *P* = 0.0340), Shannon (C vs. ABX, *P* = 0.0001; ABX vs. L, *P* = 0.0436; C vs. L, *P* = 0.0082), and Simpson (C vs. ABX, *P* = 0.0024) indices, increased the relative abundance of *Firmicutes* and *Actinobacteria* at the phylum level, increased the relative abundance of *Mannheimia* at the genus level, and made a significant division of the ABX group from the C group.

**FIGURE 6:**
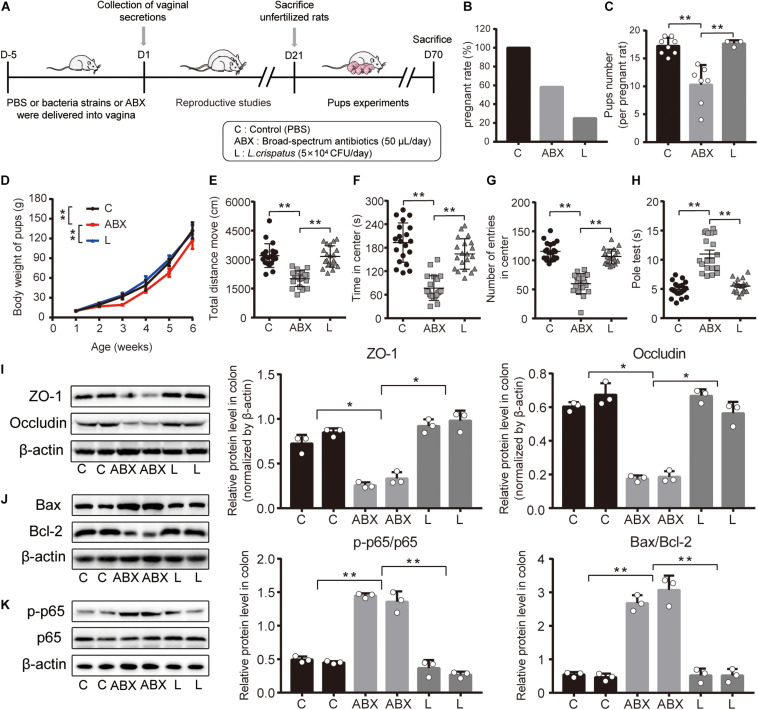
Treated vagina with *L. crispatus* had no reproductive and developmental toxicity on maternal and offspring rats. **(A)** Scheme of experimental timeline and procedures. **(B)** Fertility outcomes about pregnancy rates and **(C)** number of pups from the female rats of the indicated treatment groups. **(D)** Comparison of the body weight of pups among C, ABX, and L groups from P 7 to P 42. **(E)** Total moving distance in the open-field test. **(F)** The time of pups in center total moving distance in the open-field test. **(G)** The number of entries in the center in the open-field test. **(H)** The time of pups climbing pole in the pole test. **(I)** Expression level of intestinal tight junction proteins, ZO-1, and Occludin. **(J)** Protein expression level of apoptosis-associated factors, Bcl-2 and Bax. **(K)** Protein expression level of p-p65 and p65. The data represent means ± SD of eight to 20 animals or three independent experiments. *P < 0.05, **P < 0.01. SD, standard deviation; C, control; ABX, wide-spectrum antibiotic; L, *L. crispatus*.

Then, we compared the fertility outcomes in the C, ABX, and L groups. As shown in Figures 6A–C, the use of broad-spectrum antibiotics and *L. crispatus* both reduced the conception rates of rats (ABX vs. L, 7 vs. 3), and *L. crispatus* had more pup number after conception compared with the ABX group (ABX vs. L, 10 vs. 17, *P* = 0.0016), and no obvious difference was observed between C and L groups.

Then, we studied the effects of ABX and *L. crispatus* on offspring health and development, and our results indicated that the use of ABX greatly reduced the body weight growth from postnatal day (P) 7 to P 42 (P 42, ABX vs. L, 117.5 vs. 128.59 g) (Figure 6D). Additionally, offspring anxiety-like behavior, motor coordination, and cognitive deficits were also evaluated by open-field test and polo test, and the results showed that the pups in groups C and L moved the longer mean moving distance (C vs. ABX vs. L, 3,206.699 cm vs. 2,017.157 cm vs. 3,167.3345 cm), stayed a longer time in the center (C vs. ABX vs. L, 193.2325 s vs. 76.905 s vs. 164.08 s), significantly increased the number of entering to the center (C vs. ABX vs. L, 116 vs. 60 vs. 107), and had the lowest latency time (C vs. ABX vs. L, 4.9175 s vs. 10.9955 s vs. 5.5145 s) compared with the ABX group, respectively (Figures 6E–H). We subsequently examined the tight junction proteins, apoptosis factors, and inflammatory-related factors in the colon of pups, and we found that the ABX group exhibited lower expression levels of ZO-1 and Occludin (Figure 6I) and higher expression levels of Bax/Bcl-2 and p-p65/p65 (Figures 6J,K) compared with the C group. For the L group, it seemed that the use of *L. crispatus* would not affect offspring development, while excluded vaginal microbiota using broad-spectrum antibiotics poses a negative effect on offspring development.

## Discussion

A healthy vaginal microbiome is dominated by species of *Lactobacillus* that have a protective effect and may have therapeutic potential (Codagnone et al., 2019). As with the gut microbiome, disruption of the vaginal microbiome may affect the immunity of the body or lead to an increase in pathogens, potentially leading to the occurrence of diseases (Brown et al., 2019). Although some studies have demonstrated the relationship between bacteria and fertilization (Fredricks et al., 2005; Srinivasan et al., 2012), the underlying mechanisms involved in it is unclear. Our work indicated that *L. crispatus* not only has the sperm impairment effect *in vitro* but also may influence the maternal reproductive ability *in vivo*.

Motility is the key factor of sperm function which can be used to predict the fertilization potential of sperm *in vitro*, while the maintenance of sperm function is mainly dependent on [Ca^2+^]_*i*_ (Agnihotri et al., 2016). Our study found that *L. crispatus* greatly reduced the total sperm motility, progressive motility, linear velocity, immotile sperm, [Ca^2+^]_*i*_, and the ability of sperm to penetrate the viscous medium, which give us an insight that *L. crispatus* has the potential to affect fertilization (Figures 1, 2). It was also implying that the inhibitory effect of adhered bacteria on sperm motility and their ability to penetrate the methylcellulose medium may result from the reduction of sperm [Ca^2+^]_*i*_ *in vitro.* Nevertheless, whether *L. crispatus* has the same action *in vivo* is not clear. Therefore, the *in vivo* sperm inhibitory efficacy of bacteria was assessed using the rat model.

Fertility outcome experiments showed that female rats treated with VNP20009, VNP20009 DNase I, *E. coli* O157:H7, and *L. crispatus* had a reduction of conception rates, respectively. However, *L. crispatus* did not affect fetal development and survival. Other bacteria reduced the fetal survival rate after pregnancy and led to a dead, malformed, or absorbed fetus (Supplementary Figure 1). The V, VD, and E groups also showed a serious inflammation reaction and tissue damage. Moreover, the production of apoptosis factors (FasL) (Krzyzowska et al., 2011) was obviously detected in uterus slices, which indicated that these bacteria had reproductive toxicity, but *L. crispatus* protected the uterine health of rats (Figure 3).

Recently, apoptosis has been implicated in regulating various reproductive tissues, including the uterus, ovary, placenta, and fetal membrane (Mu et al., 2002; Mitchell et al., 2015). In addition to FasL, we found that the expressions of Bax/Bcl-2 in the L group were also significantly ameliorated compared with the other experimental groups and mainly occurred at the protein level. When we explored the body immune status, we found that the balance is disturbed, such that the TNF-α, IL-6, and IL-1β cytokines in the serum and the uterine tissues were higher in the V, VD, and E groups, which were coincident with the levels of inflammation found in uterus slices. As TNF-α, IL-6, and IL-1β are the three prominent pro-inflammatory cytokines that can activate NFκB transcription, the TLR/NFκB pathway was further studied (Joo et al., 2012). As expected, the expression of key proteins in the TLR/NFκB pathway including TLR-4 and p-p65/p65 was also significantly increased in the pathogen group, while no obvious changes were observed between the L group and the C group. Then, we also measured the expression of TGF-β1, a protein whose disordered expression in the maternal uterus might be associated with embryo implantation failure (Rajaei et al., 2011; Guo et al., 2018). We found that the uterus in the L group had normal TGF-β1 level that was consistent with the C group but which in the V, VD, and E groups were aberrant (Figure 4). The abnormal expression of TGF-β1 leads to an imbalance between apoptosis and proliferation of uterine tissues, resulting in the occurrence of abortion and other adverse pregnancy outcomes (Latifi et al., 2019). These data together suggested that the interventions of *L. crispatus* kept the uterine healthy and avoided the apoptosis and inflammation of the uterus compared with other pathogens.

Communities of microbiota have been proved to be associated with numerous health outcomes (Younes et al., 2018). We integrated vaginal microbiome datasets using the 16S rRNA gene high-throughput sequencing to reveal the functional impact of microbiome on genital health with the rat model. Regarding the vaginal microbiota at the phylum level, we observed a lower relative abundance of *Proteobacteria* and a higher relative abundance of *Firmicutes* in the C and L groups. Moreover, our results showed that VNP20009, VNP20009 DNase I, and *E. coli* O157:H7 could cause vaginal dysbiosis by downregulating *Bifidobacterium* and upregulating *Acinetobacter* (opportunistic pathogen) and *Bacteroides* at the genus level. These specific changes may contribute to the development of inflammatory-related diseases and are associated with the pathogenesis of bacterial vaginosis (Anstey Watkins et al., 2019; Figure 5).

In this study, we found that the vaginal microbiota, reproductive health, and inflammation were closely correlated. The abundance of beneficial bacteria (*Firmicutes* and *Bifidobacterium*) was negatively associated with pro-inflammatory indicators to some extent. Reversely, *Proteobacteria*, *Acinetobacter*, and *Bacteroides* were positively associated with pro-inflammatory indicators. VNP20009, VNP20009 DNase I, and *E. coli* O157:H7 did not colonize the rat vagina; they all showed a serious destruction to the maternal vaginal microbiota, leading to an imbalance in the immune environment. Although *L. crispatus* showed a potential to inhibit sperm motility, it maintained the normal vaginal microbiota homeostasis and did not adversely affect the reproductive health of rats.

Recent reports have demonstrated that the pioneer community of maternal vaginal microbiota that colonizes the offspring gastrointestinal tract at birth has a lasting impact on the gut and brain (Peterson and Artis, 2014; Sakwinska et al., 2017; Jasarevic et al., 2018). Our results demonstrated that broad-spectrum antibiotics greatly reduced the maternal vaginal microbiota diversity and overall abundance (Supplementary Figure 4). In addition, the rats transplanted with broad-spectrum antibiotics exhibited an intermediate inhibitory effect of conception and pup number, and their offspring also had a lower body weight curve, anxiety-like behavior, uncoordinated movement, and cognitive impairment, indicating that the use of antibiotics not only greatly reduced the vaginal microbiota but also posed a serious negative effect on offspring health. The experiments on the intestinal health of the offspring have also shown that the colonic tight junction (TJ) proteins (Feng et al., 2019) – ZO-1 and Occludin – were reduced in the ABX group, and the expression of inflammation factors (p-p65/p65) and apoptosis factors (Bax/Bcl-2) in the colon tissue was also increased in the ABX group, which suggested that the intestinal health of offspring from the ABX group was at risk (Figure 6). In summary, *L. crispatus* could sustain the maternal vaginal composition and had no effect on the healthy development during the early life of the offspring as the normal female rats.

In obstetrics and gynecology, *Lactobacillus*-based probiotics have been used to restore the physiologic vaginal microbiota to alter the maternal and neonatal microbiome (Czaja et al., 2007; Srikrishna and Cardozo, 2013), thus improving pregnancy and neonatal outcomes (Stone, 2018). Our study found that *L. crispatus* protected vaginal health and offspring development, which provided the foundation for vaginal microbiome transplantation, and also gave a theoretical basis for the development and application of *L. crispatus* as a vaginal probiotic product in the future. However, the limitations of *L. crispatus* in inhibiting sperm motility also provide challenges, including achieving a stable colonization of the desired probiotic species, avoiding over-transplanting of probiotic species in the face of intrinsic and extrinsic vaginal disturbances, as well as determining the ideal species or strains for different women individuals.

## Conclusion

In the present study, we evaluated the adhesion ability of *L. crispatus* Lcr-MH175 to sperm. All bacteria could adhere to sperms effectively and reduce their motility, which were particularly obvious for the probiotic *L. crispatus*. This property of *L. crispatus* reduced the pregnancy rate in a rat reproduction model but did not affect maternal health or offspring development. We postulate that the reduction in motility caused by the adhesion of *L. crispatus* may be beneficial for healthy couples and offspring health as it could reduce the chance of a poor-quality sperm combining with the egg and provide a unique sperm selection mechanism. Additionally, the strong adherent effect of *L. crispatus* may be detrimental for men with severe asthenospermia, oligospermia, or aspermia as *L. crispatus* could lower the potential of the sperm combining with the egg. Therefore, although *L. crispatus* is a probiotic species, more caution should be paid when using it as a vaginal viable preparation for women of child-bearing age, such as the dosage. Taken together, this study focuses exclusively on the role of *L. crispatus* on sperm activity and the development of offspring, so more work is needed to explore the mechanistic insights of *L. crispatus* and other *Lactobacillus* spp. on sperm functions.

## Data Availability Statement

The datasets presented in this study can be found in online repositories. The names of the repository/repositories and accession number(s) can be found in the article/Supplementary Material.

## Ethics Statement

The animal study was reviewed and approved by the Laboratory Animal Ethics Committee of Nanchang Royo Biotech Co., Ltd. (RYE2019040801).

## Author Contributions

PL contributed to the methodology, investigation, formal analysis, visualization, and writing – original draft. KW contributed to the methodology, investigation, and visualization. XH contributed to the methodology. LZ and JW contributed to the formal analysis. ZL and XC contributed to the investigation. HW contributed to the resources and conceptualization. TC contributed to the conceptualization, funding acquisition, supervision, and writing – review and editing. All authors contributed to the article and approved the submitted version.

## Conflict of Interest

The authors declare that the research was conducted in the absence of any commercial or financial relationships that could be construed as a potential conflict of interest.

## Publisher’s Note

All claims expressed in this article are solely those of the authors and do not necessarily represent those of their affiliated organizations, or those of the publisher, the editors and the reviewers. Any product that may be evaluated in this article, or claim that may be made by its manufacturer, is not guaranteed or endorsed by the publisher.
