# Supplementary material for: Vaginal Probiotic Lactobacillus crispatus Seems to Inhibit Sperm Activity and Subsequently Reduces Pregnancies in Rat
Source: Front Cell Dev Biol. 2021 Aug 13;9:705690. doi: 10.3389/fcell.2021.705690 (PMC8414900; doi:10.3389/fcell.2021.705690)
Supplement: Supplementary file 1 [file Data_Sheet_1.pdf]

Vaginal Probiotic *Lactobacillus crispatus* With the Dual Role of Inhibiting Sperm Activity and Reducing Pregnancies

Ping Li [1], Kehong Wei [1], Xia He [2], Lu Zhang [1], Zhaoxia Liu [3], Jing Wei [1], Xiaomei Chen [1], Hong Wei [4], Tingtao Chen [1*]*

[1] School of Life Sciences, Institute of Translational Medicine, Nanchang University, Nanchang, China, [2] Department of Obstetrics and Gynecology, The Ninth Hospital of Nanchang, Nanchang, China, [3] Department of Obstetrics and Gynecology, The Second Affiliated Hospital of Nanchang University, Nanchang, China, [4] Precision Medicine Institute, The First Affiliated Hospital, Sun Yat-sen University, Guangzhou, China

[*]***Corresponding:***

Tingtao Chen, Institute of Translational Medicine, Nanchang University, Nanchang 330031, PR China; E-mail: chentingtao1984@163.com

Hong Wei, Precision Medicine Institute, The First Affiliated Hospital, Sun Yat-sen University, Guangzhou 510080, PR China; E-mail: weihong63528@163.com

Supplementary Figure 1 | The effect of the tested bacteria on the fetal health. **(A)** The number of living fetus. **(B)** The number of dead fetus. **(C)** The number of absorbed fetus. **(D)** The number of malformed fetus. The data represent means ± SD of 8-10 animals. *P < 0.05, **P < 0.01. SD, standard deviation; C, control; V, VNP20009; VD, VNP20009 DNase I; E, *E. coli* O157:H7; L, *L. crispatus*.

Supplementary Figure 2 | The number of microbial groups contained in different samples at different taxonomic levels.

Supplementary Figure 3 | Microbial diversity in the C, V, VD, E and L groups. **(A)** linear discriminant analysis effect size analysis. **(B)** Metastats analysis. LDA, linear discriminant analysis; C, control; V, VNP20009; VD, VNP20009 DNase I; E, *E. coli* O157:H7; L, *L. crispatus.*

Supplementary Figure 4 | The effect of *L. crispatus* and broad-spectrum antibiotics on the vaginal microbiota diversity of rats. **(A)** The observed species; **(B)** the Shannon index; **(C)** the Simpson index. **(D)** Microbial composition at the phylum level. **(E)** Microbial composition at the genus level. **(F)** Venn diagram of the vaginal microbiota among C, ABX, L groups. **(G)** PCoA of vaginal bacterial communities among C, ABX, L groups. The data represent means ± SD of 4 animals. *P < 0.05, **P < 0.01. SD, standard deviation; C, control; ABX, wide-spectrum antibiotic; L, *L. crispatus*; PCoA, Principle coordination analysis.
