# Supplementary figures and images for: Vaginal Probiotic Lactobacillus crispatus Seems to Inhibit Sperm Activity and Subsequently Reduces Pregnancies in Rat
Source: Front Cell Dev Biol. 2021 Aug 13;9:705690. doi: 10.3389/fcell.2021.705690 (PMC8414900; doi:10.3389/fcell.2021.705690)

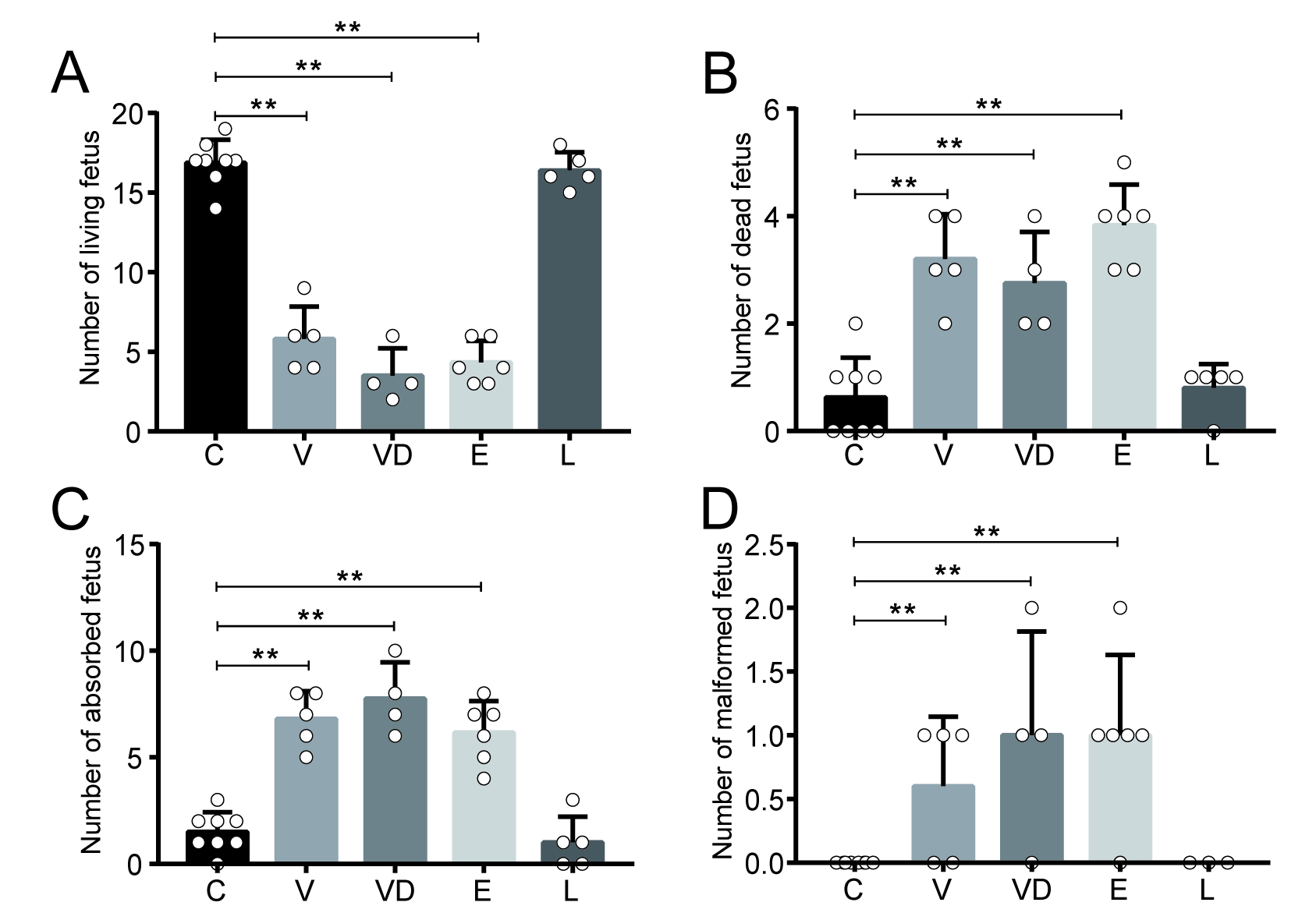

Supplement: Supplementary file 2 [file Image_1.TIF]

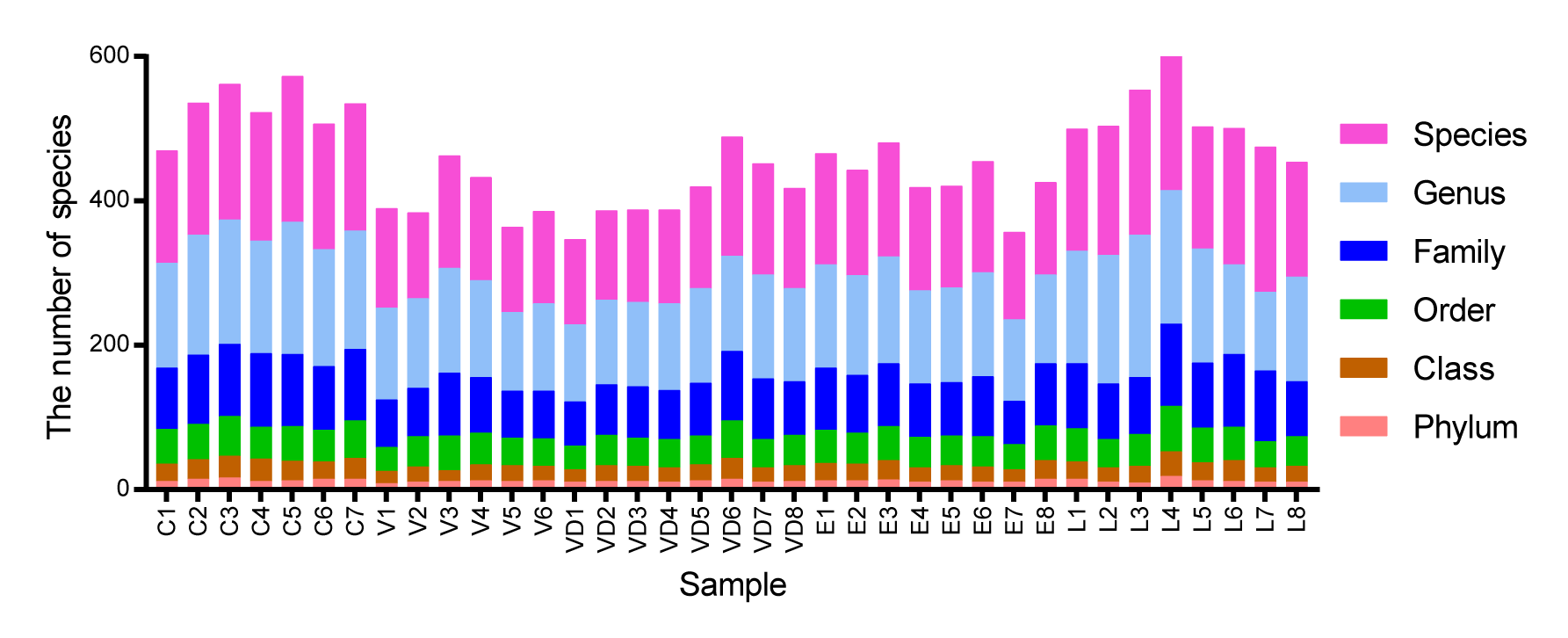

Supplement: Supplementary file 3 [file Image_2.TIF]

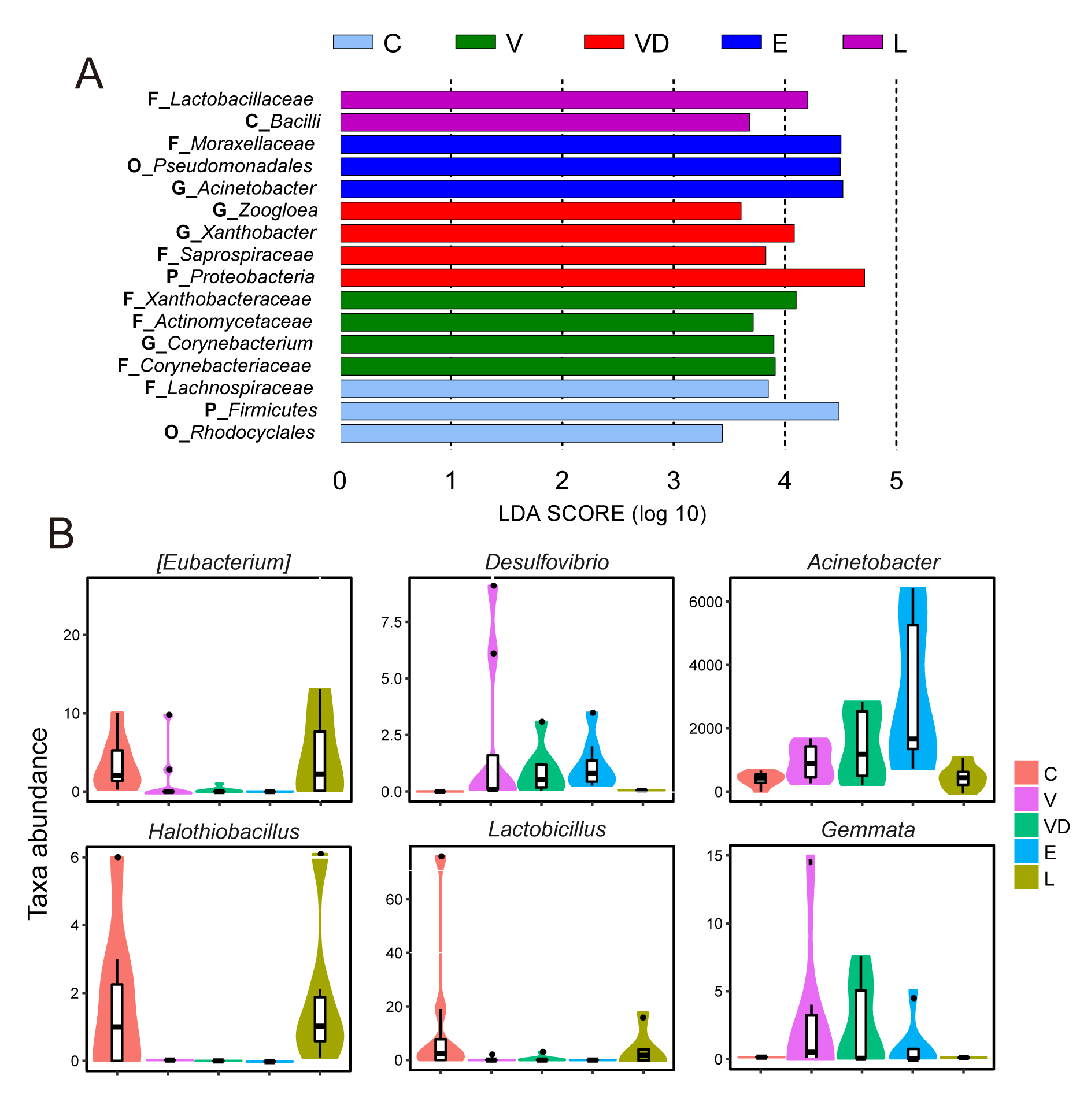

Supplement: Supplementary file 4 [file Image_3.TIF]

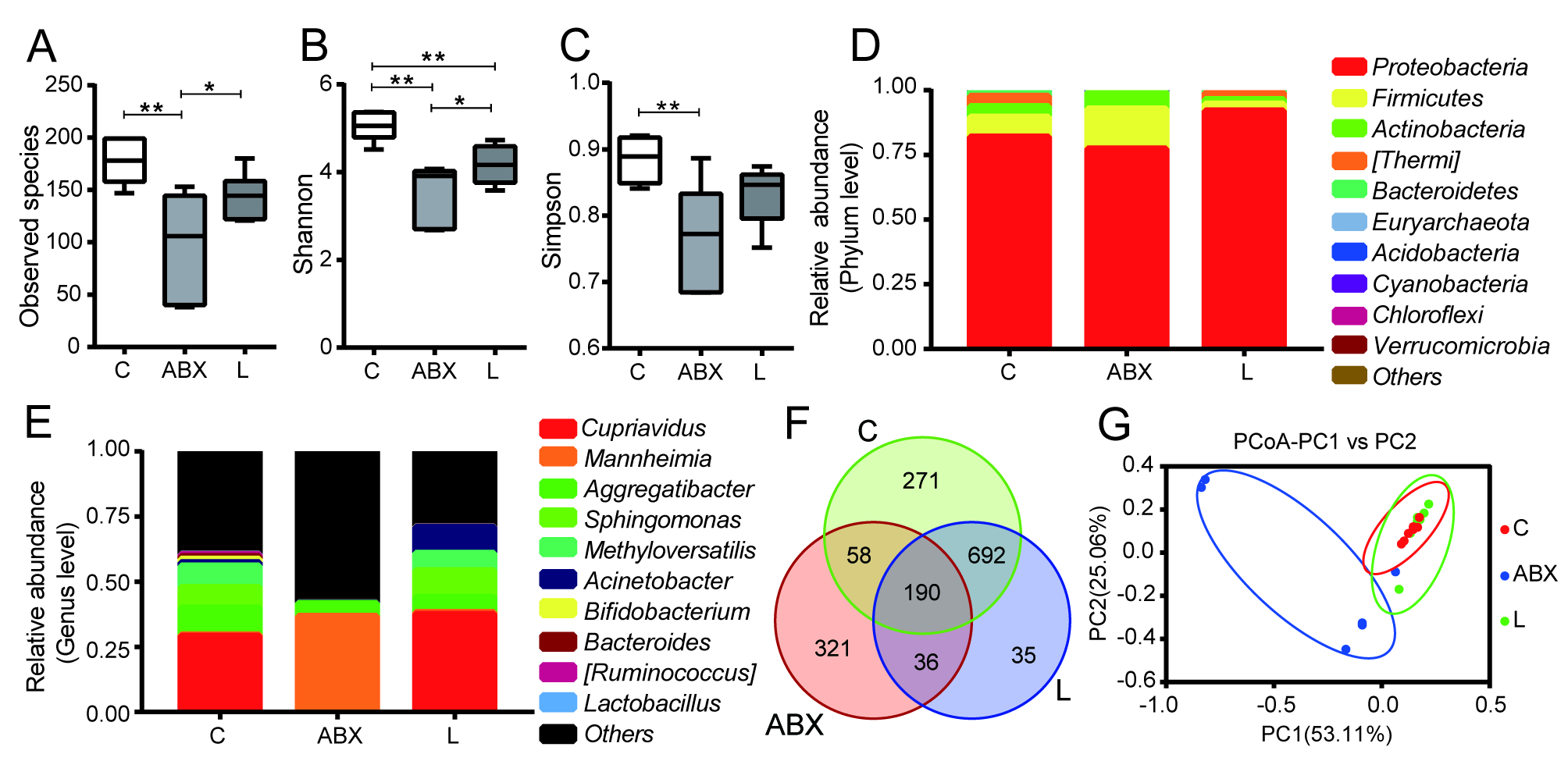

Supplement: Supplementary file 5 [file Image_4.tif]
